# In this month’s Bulletin

**DOI:** 10.2471/BLT.24.000224

**Published:** 2024-02-01

In the editorial section, Viroj Tangcharoensathien et al. (86) introduce this special issue on geopolitics, global health and equity for the 2024 Prince Mahidol Award Conference. Michelle McIsaac and Felicia Marie Knaul (87) discuss how gender gaps in employment and compensation affect health equity.

Gary Humphreys (90–91) reports on efforts to mitigate climate risks in informal urban settlements. Mirai Chatterjee (92–93) talks to Fid Thompson about the social determinants of health that affect women working in the informal sector.

## Afghanistan, Algeria, Angola, Bangladesh, Benin, Burundi, Cameroon, Central African Republic, Chad, Côte d’Ivoire, Democratic Republic of the Congo, Dominican Republic, Gambia, Ghana, Guinea, Guinea-Bissau, Haiti, Honduras, India, Indonesia, Iraq, Kiribati, Lao People´s Democratic Republic, Liberia, Madagascar, Malawi, Mali, Mauritania, Mozambique, Myanmar, Nepal, Nigeria, Pakistan, Papua New Guinea, Paraguay, Peru, Philippines, Rwanda, Sao Tome and Principe, Senegal, Sierra Leone, Timor-Leste, Togo, Uganda, United Republic of Tanzania, Zambia 

### Factors associated with coverage of maternal health interventions

Leonardo Z Ferreira et al. (105–116) present a composite index for socioeconomic deprivation.

## Australia

### Indigenous health

Abdullah A Mamun et al. (146–148) call for research on inequalities.

## Global 

### International investment agreements

Takwa Tissaoui et al. (94–104) draw out health policy implications of corporate social responsibility provisions.

### Global distribution of health workers

Margaret Walton-Roberts and Ivy L Bourgeault (117–122) explore policies that govern international migration.

### Global health security

Clare Wenham (123–129) details how forum shifting affects negotiations. 

### Decolonizing global health

David McCoy et al. (130–136) discuss ways to improve equity and ensure justice. 

### Global health initiatives 

Shams El Arifeen et al. (137–139) offer four ways to improve evaluation methods and relevance.

### Self-care interventions

Manjulaa Narasimhan et al. (140–142) outline ways to mitigate inequities in health coverage.

### Migration, health care and health outcomes

Elisa Mosler Vidal and Kolitha Prabash Wickramage (143–245) show that health data needs to be disaggregated for migrant status.

